# Exploring the burdens of women living with Fabry disease in Japan: A patient survey of 62 respondents

**DOI:** 10.1016/j.ymgmr.2025.101231

**Published:** 2025-05-30

**Authors:** Masahisa Kobayashi, Ikuko Kaku, Nanae Goto, Mio Tsuchiya, Norio Sakai

**Affiliations:** a Department of Pediatrics, The Jikei University School of Medicine, 3-25-8 Nishi-Shinbashi, Minato-ku, Tokyo 105-8461, Japan; b Japan Fabry Disease Patients and Family Association (JFA), 305 Shuwa Shinsakamachi Residence, 8-5-9 Akasaka, Minato-ku, Tokyo 107-0052, Japan; c Patient & Professional Advocacy, Amicus Therapeutics K.K., Shin Marunouchi Center Building 19F, 1-6-2 Marunouchi Chiyoda-ku, Tokyo 100-0005, Japan; d Center for Promoting Treatment of Intractable Diseases, ISEIKAI International General Hospital, Osaka 530-0052, Japan

**Keywords:** Fabry disease, Women, Quality of life, Patient survey, Japan, Mental burden

## Abstract

The challenges encountered by women living with Fabry disease in Japan are not well understood. This study aimed to elucidate the experiences of women with Fabry disease and their support networks from both female and male perspectives. A 22-question survey was conducted among patients with Fabry disease and their caregivers (≥18 years) in Japan between August and October 2023. Sixty-two recipients completed the questionnaire (11.5 % response rate); 47 (75.8 %) were female and the mean age was 52.4 years. Overall, 51 respondents (82.3 %) identified as patients, 2 (3.2 %) as caregivers, 6 (9.7 %) as both a patient and caregiver, and 3 (4.8 %) as “other”. In total, 43 respondents (69.4 %) were women with Fabry disease. Among life events surveyed, Fabry disease had the greatest impact for women during family planning. The most commonly reported concerns for women were inheritance of Fabry disease and impact on children, the main reasons for which were prejudice, stigma, and sense of guilt associated with inheritance. In all, 28.1 % of respondents felt family and colleagues understood women's challenges with Fabry disease, while 37.9 % believed their primary care physicians and 48.3 % felt their specialist physicians understood these challenges; 26.3 % thought women received tailored care, and 75.9 % felt the condition affects mental health. Women with Fabry disease in Japan face substantial emotional burdens and lack support from their community and physicians. Healthcare professionals can play a pivotal role by offering genetic counseling and developing support programs to alleviate mental burdens and provide education about the disease and family planning implications.

## Highlights

• The challenges encountered by adult women living with Fabry disease and their caregivers were examined using a 22-question survey.
• Participants reported that the key event in a woman’s life that is most affected by Fabry disease is family planning.
• The concerns most frequently held by women living with Fabry disease were reported to be inheritance and impact on children.
• Over three-quarters of respondents agreed that the disease affects women’s mental health.

## 1 Introduction

Fabry disease is a rare, chronic, progressive, X-linked lysosomal disorder caused by variants in the galactosidase alpha (*GLA*) gene leading to a deficiency in α-galactosidase A enzyme activity [1]. This deficiency results in the progressive accumulation of glycosphingolipids, primarily globotriaosylceramide (Gb_3_) and globotriaosylsphingosine (lyso-Gb_3_), within the lysosomes of various cell types, including those with particular relevance to disease pathology (e.g. vascular endothelial cells, cardiomyocytes, renal cells, and neurons) [1,2]. As a result of this cellular dysfunction, individuals with Fabry disease experience a range of symptoms, including pain, early strokes, cardiovascular complications, gastrointestinal symptoms, and renal failure, which left untreated substantially limit life expectancy [[2], [3], [4]].

Historically, Fabry disease was thought to primarily affect males because of its X-linked inheritance pattern, with heterozygous females mistakenly considered asymptomatic carriers [5,6]. However, it is now recognized that women with a single pathogenic variant in the *GLA* gene can exhibit a wide range of clinical symptoms, sometimes as severe as those in affected males [[5], [6], [7]].

In Japan, Fabry disease is classified as an intractable disease [8]; however, significant pharmacological advancements over the past three decades have led to effective treatments that address the deficiency of α-galactosidase A [[9], [10], [11]]. Enzyme replacement therapy (ERT) and pharmacological chaperone therapy are the primary treatment strategies, aiming to prevent multiorgan involvement and improve patient outcomes [12]. ERT involves the intravenous administration of recombinant enzymes, which help reduce the accumulation of Gb_3_ in lysosomes [9,12]. Pharmacological chaperone therapy, with the oral agent migalastat, stabilizes the enzyme, increasing its trafficking to the lysosome and consequently enhancing its activity [10,12]. Additionally, emerging therapies such as plant-derived ERTs, substrate reduction therapies, and gene therapy are under investigation, offering promising advancements in the management of Fabry disease [12].

The estimated birth prevalence of Fabry disease is 1 in 40,000 to 1 in 170,000 [13]. However, newborn screening suggests a higher prevalence, with estimates of 1 in 11,854 newborns carrying known pathogenic *GLA* variants in Japan [14], and ∼1 in 1250 newborn males with the disease in Taiwan [15]. There are thought to be around 1600 patients living with Fabry disease in Japan [16]. Over two-thirds of heterozygous women develop symptoms, with one in five experiencing major cerebrovascular, cardiac, or renal events, at a median age of 46 years [17,18]. The phenotypic diversity exhibited by women is higher than in men, with symptoms typically appearing later and progressing gradually [19]. Female patients face a distinct set of challenges, including delayed diagnosis and under-recognition of symptoms, due in part to the incorrect perception that they are merely carriers [18,19]. Studies also indicate that heterozygous women often encounter negative experiences with healthcare providers because of the providers' lack of knowledge and the disease's complexity [20].

Women's overall quality of life (QoL) can be significantly affected by the multifaceted symptoms of Fabry disease, which impact many aspects of physical, emotional, and social wellbeing [4,21,22]. While ERT has shown considerable benefits in slowing disease progression, the burden of frequent infusions and potential side effects can affect QoL further [20,22]. Despite advancements, there remains a critical need for a deeper understanding of the specific challenges faced by women with Fabry disease to help enhance patient care and support. Notably, there is an absence of published data focusing on QoL for women living with Fabry disease in Japan.

This study aims to explore the burden of disease and QoL in women living with Fabry disease in Japan, highlighting their unique experiences and unmet needs through the perspectives of patients, caregivers, and supporters of both male and female gender. By examining the impact of Fabry disease on physical, emotional, and social wellbeing, this study seeks to inform healthcare providers and researchers about the importance of sex-specific considerations in the management of Fabry disease, ultimately contributing to better health outcomes and improved QoL for affected women.

## 2 Material and methods

### 2.1 Study design

A non-interventional study was conducted from August to October 2023 to explore the potential burden of Fabry disease in women in Japan.

Patients with Fabry disease and/or their caregivers who were at least 18 years of age and able to give consent were eligible to participate in the survey (Supplementary materials). At the suggestion of JFA members, male patients and/or caregivers were included to gather their perspectives and to help promote better understanding of women's experiences with Fabry disease among men, as their involvement is considered essential in addressing women's unmet needs, such as mental health challenges and family planning concerns. All participants (whether female, male, a patient, or a caregiver) were asked to answer questions on the challenges faced by women living with Fabry disease to provide diverse perspectives on this issue. Women living with Fabry disease were defined as mothers, wives, daughters, or the participant themselves as a patient or caregiver. The protocol and questionnaire were developed by Amicus Therapeutics K.K. (Tokyo, Japan) and the Japan Fabry Disease Patients and Family Association (JFA). The questionnaire comprised 22 questions relating to demographics, disease history, symptoms, treatment status, and the challenges faced by women with Fabry disease (Supplementary materials).

To protect patient privacy, the JFA determined the number of questionnaires sent to each household and the survey was administered by Macromill Carenet, Inc. (Tokyo, Japan), a third-party healthcare research organization. A total of 540 paper-based questionnaires were distributed by mail to 180 registered families. The front page of the questionnaire included a quick response (QR) code and a web link, giving participants the option to complete the survey online. To prevent duplication, each respondent was assigned a unique ID. Completed paper questionnaires were returned anonymously by post to the study office.

### 2.2 Ethics

The survey was conducted in accordance with the globally accepted ethical principles of the Declaration of Helsinki and Good Clinical Practice guidelines, and in keeping with local regulations. The questionnaire and protocol were reviewed and approved by the ethics committee of Yokohama Minoru Clinic. Written, informed consent was obtained from respondents prior to participation.

### 2.3 Statistical analysis

The analyses were performed using anonymized data. Descriptive statistics and Fisher's exact test were employed, with *p* < 0.05 considered significant. Thematic analysis of open-ended responses was conducted using an inductive approach, yielding themes and sub-themes [23], and co-occurrence was evaluated using the Jaccard index, with values of at least 0.20 being represented by circles and lines.

## 3 Results

### 3.1 Participant demographics and characteristics

A total of 62 questionnaires (11.5 %) were completed, returned, and included in the analysis. The mean age of respondents was 52.4 years and 75.8 % were female (Table 1). The most common occupations were full-time company/public/organization employee (33.9 % of respondents), full-time homemaker (25.8 %), and part-time job (19.4 %). Overall, 51 respondents (82.3 %) classified themselves as a patient with Fabry disease; two (3.2 %) as a caregiver for someone with the condition and six (9.7 %) as both a patient and a caregiver; of the remaining three patients (4.8 %) who selected “other”, two reported that they were a family member of a patient and one did not provide a further description of their status.

**Table 1:** Participant demographics.

	Overall	Sex	
	(*N* = 62)	Female (*n* = 47)	Male (*n* = 15)
Sex, *n* (%)			
Female	47 (75.8)	–	–
Male	15 (24.2)	–	–
Age, years			
Mean (SD)	52.4 (15.0)	53.4 (15.8)	49.3 (12.2)
Median (range)	50 (19–77)	50 (19–77)	48 (31–72)
Occupation, *n* (%)			
Student	2 (3.2)	2 (4.3)	0
Company/public/organization employees	21 (33.9)	13 (27.7)	8 (53.3)
Self-employed or freelance	4 (6.5)	3 (6.4)	1 (6.7)
Full-time homemaker	16 (25.8)	16 (34.0)	0
Part-time job	12 (19.4)	11 (23.4)	1 (6.7)
Unemployed	5 (8.1)	1 (2.1)	4 (26.7)
Other	2 (3.2)	1 (2.1)	1 (6.7)
Patient/caregiver, *n* (%)			
Patient	51 (82.3)	37 (78.7)	14 (93.3)
Patient and caregiver	6 (9.7)	6 (12.8)	0
Caregiver	2 (3.2)	2 (4.3)	0
Caring for a child	7 (11.3)	7 (14.9)	0
Caring for a spouse or partner	1 (1.6)	1 (2.1)	0
Other	3 (4.8)	2 (4.3)	1 (6.7)

SD, standard deviation.

**Fig. 1:**
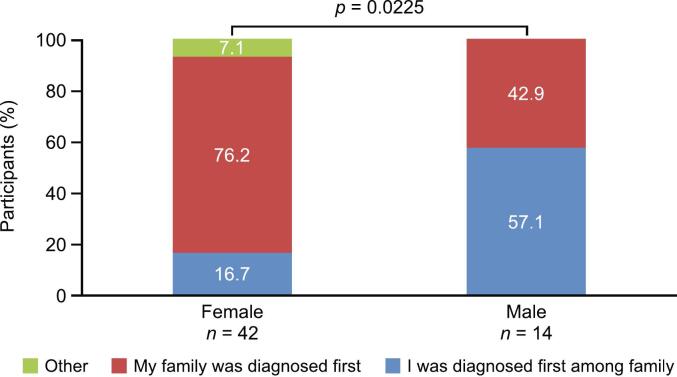
Diagnosis of Fabry disease.

Significantly more male than female patients were the first in their family to receive a diagnosis of Fabry disease (57.1 % and 16.7 %, respectively; *p* = 0.0225; Fig. 1). A further 7.1 % of female patients, and no male patients, were diagnosed simultaneously with a family member or were aware of a wider family history of the disease. The mean age at diagnosis was 37.7 years for women and 30.5 years for men (Table 2).

**Table 2:** Age at diagnosis of Fabry disease.

	Overall	Sex	
	(*N* = 57)	Female (*n* = 43)	Male (*n* = 14)
Mean age at diagnosis (SD), years	35.9 (14.9)	37.7 (14.5)	30.5 (15.3)
Median (range) age at diagnosis, years	37 (7–70)	38.5 (7–70)	30 (9–58)
Age at diagnosis, years, *n* (%)			
0–9	3 (5.3)	1 (2.3)	2 (14.3)
10–19	5 (8.8)	2 (4.7)	3 (21.4)
20–29	10 (17.5)	8 (18.6)	2 (14.3)
30–39	13 (22.8)	11 (25.6)	2 (14.3)
40–49	15 (26.3)	11 (25.6)	4 (28.6)
50–59	8 (14.0)	7 (16.3)	1 (7.1)
60–69	1 (1.8)	1 (2.3)	0
70–79	1 (1.8)	1 (2.3)	0
Unknown	1 (1.8)	1 (2.3)	0

### 3.2 Symptoms and treatment

Most Fabry disease patients reported currently experiencing symptoms of the disease (86.0 % of female and 92.9 % of male patients; Table 3). Most female patients (88.4 %) and all male patients reported that they were currently receiving treatment, the most common being ERT (69.8 % of female and 85.7 % of male patients). Among the five female patients not receiving treatment, four gave the reason that their primary physician told them that treatment was not needed, while two of the five reported that they personally held this view.

**Table 3:** Fabry disease treatment.

	Overall	Sex	
	(*N* = 57)	Female (*n* = 43)	Male (*n* = 14)
Patients currently experiencing symptoms of Fabry disease, *n* (%)	50 (87.7)	37 (86.0)	13 (92.9)
Patients currently receiving treatment for Fabry disease, *n* (%)	52 (91.2)	38 (88.4)	14 (100.0)
ERT, *n* (%)	42 (73.7)	30 (69.8)	12 (85.7)
Pharmacological chaperone therapy, *n* (%)	10 (17.5)	8 (18.6)	2 (14.3)
Patients not currently receiving treatment for Fabry disease, *n* (%)	5 (8.8)	5 (11.6)	0 (0.0)
Reasons for not receiving treatment (multiple answers permitted), *n*			
I don't have time because of work commitments	1	1	0
I don't have time because of family commitments	1	1	0
Financial burden (cost of medical care and transportation to the hospital)	0	0	0
I don't think it's a problem that needs treatment	2	2	0
I was told by my primary physician that there was no need for treatment	4	4	0
Communication with the primary physician is a psychological burden	0	0	0
Communication with the medical staff is a psychological burden	0	0	0
Other	1	1	0

**Fig. 2:**
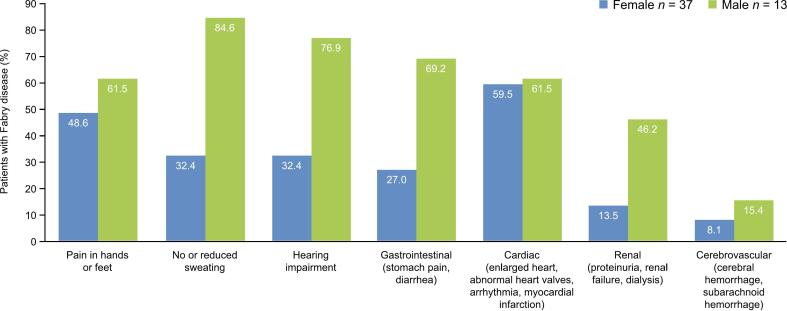
Current symptoms and conditions related to Fabry disease.

Patients who reported having symptoms selected all that they were currently experiencing from a list of seven manifestations. The most frequent among all respondents were cardiac symptoms (60.0 %), pain in hands or feet (52.0 %), and no or reduced sweating (46.0 %). All seven symptoms were reported in a higher proportion of men than women (Fig. 2). The most common current symptoms reported by male patients were no or reduced sweating (84.6 %), hearing impairment (76.9 %), and gastrointestinal symptoms (69.2 %), while female patients most frequently reported cardiac symptoms (59.5 %), pain in hands or feet (48.6 %), no or reduced sweating (32.4 %), and hearing impairment (32.4 %).

### 3.3 Experiences of women living with Fabry disease

Male and female participants ranked the impact of Fabry disease on seven key events in a woman's life (the onset of puberty; the menstrual cycle; planning a family, marriage, and childbirth; perimenopause and menopause; the postmenopausal period; relationship building [e.g. when entering school or finding a job]; and other). The events most frequently identified as being most impacted by Fabry disease were family planning (48.4 % of respondents), the onset of puberty (17.7 %), and the menstrual cycle (12.9 %). In a subgroup analysis of responses by female patients with Fabry disease only (*n* = 43), excluding male participants and caregivers, family planning remained the most frequently impacted domain but increased to 60.5 % of respondents, while puberty onset (16.3 %) and menstrual cycle (11.6 %) maintained their positions as second- and third-most impacted events.

**Fig. 3:**
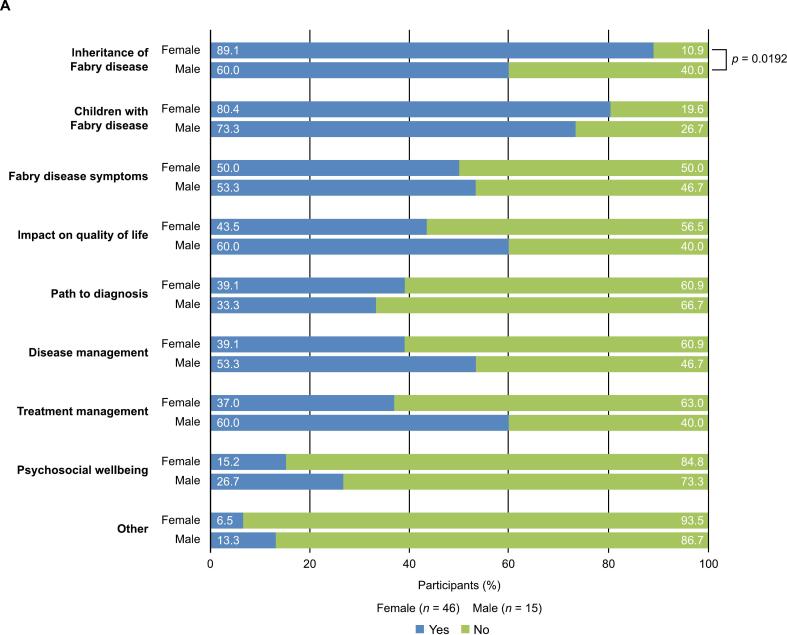

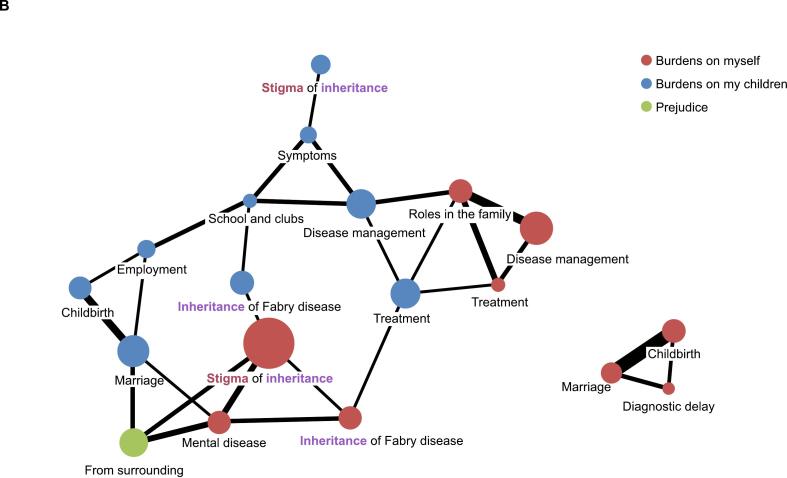
The greatest concerns for women living with Fabry disease. A) Frequency of concerns chosen by male and female respondents and B) Co-occurrence network analysis of the reasons behind the concerns chosen by male and female respondents.

Respondents chose the greatest concerns for women living with Fabry disease by selecting all applicable options from a list of nine. Both male and female respondents were asked to complete the question, and the term “women living with Fabry disease” was defined as encompassing mothers, wives, daughters, and respondents themselves as patients and family caregivers. The most frequently identified concerns were the inheritance of Fabry disease and its impact on children; notably, significantly more women than men identified inheritance as the greatest concern (89.1 % vs 60.0 %; *p* = 0.0192; Fig. 3A). Participants both male and female elaborated on the reasons that women hold these concerns through free-text responses, and thematic analysis revealed that common threads included the prejudice, stigma, and sense of guilt associated with the hereditary nature of the disease (Fig. 3B).

Larger circles indicate themes that were identified by patients at higher frequency; thicker lines represent stronger co-occurrence of themes.

**Fig. 4:**
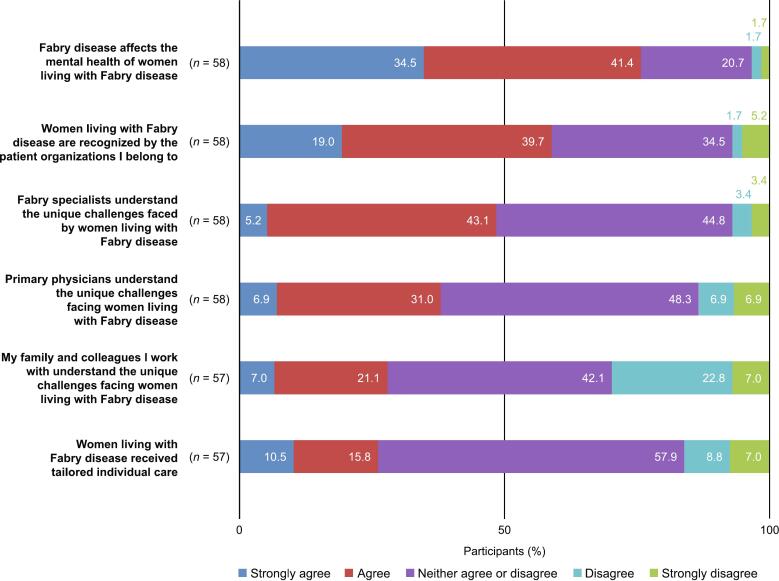
The medical and social environment for women living with Fabry disease in Japan.

Participants indicated their agreement with six statements about the medical and social environment for women living with Fabry disease in Japan, using a 5-point scale from “strongly agree” to “strongly disagree”. Overall, 75.9 % of respondents agreed or strongly agreed that Fabry disease affects female patients' mental health (Fig. 4). Over half of respondents (58.6 %) agreed or strongly agreed that patient organizations recognized their challenges. A total of 37.9 % of respondents agreed or strongly agreed that their primary physician understood the unique challenges faced by women living with Fabry disease; this figure increased to 48.3 % for Fabry disease specialist physicians. Under a third of respondents agreed or strongly agreed that their family or colleagues understood the unique challenges faced by this patient group (28.1 %), or that women living with Fabry disease received tailored individual care (26.3 %).

## 4 Discussion

This study provides important insights into the experiences of women living with Fabry disease in Japan, emphasizing the multifaceted challenges they face, based on the perspectives of patients, caregivers, and supporters of both male and female gender. The findings underscore the need for a nuanced understanding of the disease, particularly regarding the phenotypic diversity of women's symptoms and the psychosocial burdens associated with the condition.

Our findings reinforce the growing understanding that heterozygous women face dual challenges: a spectrum of physical symptoms and a significant psychological burden. Among female respondents with Fabry disease, 86.0 % reported experiencing symptoms compared with 92.9 % of males with the condition. This finding is consistent with observations from the Fabry Outcome Survey, a pivotal European database analysis [5], and a natural history study conducted in Japan, which similarly documented significant symptomatology in female patients [24]. The pronounced impact on mental health observed in our survey likely stems from multiple pathophysiological mechanisms. These include both direct neurological manifestations (cerebrovascular events, white matter lesions) and indirect psychological consequences stemming from chronic pain, fatigue, and overall disease burden [6,25,26]. While determining precise causality is challenging, our data provide compelling evidence that mental health considerations warrant particular attention in the clinical management of women with Fabry disease. Collectively, these results challenge the outdated perception of women as passive carriers and underscore the necessity for healthcare providers to recognize and address the full spectrum of symptoms experienced by female patients.

Our results provided important insights about potential gaps in communication and understanding between those directly experiencing Fabry disease and observers. The subgroup analysis conducted in this study revealed that female patients ranked family planning highest among impacted life events more frequently than the study population as a whole. In addition, fewer than half of the respondents agreed that either primary or specialist physicians understood the unique challenges faced by women with Fabry disease, highlighting a critical need for improvements in patient–physician communication and increased medical education about Fabry disease in Japan. These gaps were further evidenced in treatment decisions: among the five women not receiving treatment, four cited their physician's assessment that treatment was not needed, yet only two of the five personally held this view. These results accord with previous findings that female patients with Fabry disease may have unsatisfying experiences with healthcare professionals, arising from the triple disadvantages of disease rarity, perceived carrier status, and sex [27]. While healthcare providers may underestimate the disease impact on women, patients and caregivers also need education to understand that not every symptom is necessarily attributable to Fabry disease. Enhanced understanding among healthcare providers is crucial to facilitate tailored care and support the unique needs of female patients [25,27]. Educational initiatives should focus on symptom variability and potential disease severity in women, ensuring that healthcare professionals are equipped to provide comprehensive and holistic care. This approach will aid early diagnosis, appropriate management, and improved QoL for affected individuals.

The study sheds light on the social burdens that Japanese women living with Fabry disease endure. Beyond the medical challenges, these women reported facing a lack of understanding from family and colleagues as well as prejudice, societal stigma, and a sense of guilt owing to the hereditary nature of the disease. The findings are consistent with previous studies in which women with Fabry disease reported feelings of guilt for passing the disease on to their children, coupled with anxiety or depression, and exacerbated by a sense that they are stigmatized by society [20,21]. In a previous Japanese survey, most women being treated for Fabry disease felt their daily life was restricted by the condition and experienced difficulties in their relationships with those around them [22]. Addressing stigma and prejudice requires a concerted effort to educate communities about the genetic aspects of Fabry disease and to promote acceptance and support for affected individuals.

Healthcare professionals play a pivotal role in alleviating the mental burdens associated with Fabry disease [25,27,28]. By utilizing genetic counseling resources and developing patient support programs they can provide patients and their families with a clearer understanding of the disease's hereditary nature and its implications for family planning [27,28]. These programs should be designed to empower women with Fabry disease, offering them the tools and support needed to navigate the challenges they face and improve their overall wellbeing.

### 4.1 Study limitations

While the survey offers valuable insights, several limitations must be acknowledged. The response rate of 11.5 % was low, possibly reflecting survey fatigue among patients who may have previously participated in multiple questionnaires, and because mail distribution had limitations compared with web-based or in-person methods. Representation was particularly low among unaffected family members (*n* = 2) and there was a gender imbalance among respondents (47 women vs 15 men), suggesting perceived irrelevance, disengagement, or insufficient knowledge regarding women's Fabry disease experiences among some caregivers and men. A more balanced distribution among participant categories may have strengthened the findings. Nonetheless, this study represents the highest participation to date in a Japanese Fabry disease survey [22].

The heterogeneity within the study population (women, men, patients, and caregivers) enriches the data but complicates interpretation. In the questionnaire, “women living with Fabry disease” was defined broadly to include both female patients and female household members affected indirectly. While this inclusive approach captures a comprehensive view of disease impact, it potentially obscures differences between patient experiences or needs and those of family members or caregivers.

Another limitation may be the over-representation of female patients who are receiving treatment for Fabry disease (88.4 %), which likely exceeds the treatment rate in the broader Japanese female Fabry population, though data are lacking. This over-representation may stem from questionnaire distribution through the JFA, whose members may have heightened disease awareness, while the respondents' age could have also influenced results, as older patients typically exhibit greater awareness and specialist care-seeking behavior. These factors should be considered when interpreting study findings.

Despite these limitations, the survey provides valuable insights into this population of Japanese women living with Fabry disease. The data underscore the need for a patient-centered approach that considers the unique challenges faced by women with the condition. By highlighting the lived experiences of these patients and caregivers, the study contributes to a deeper understanding of Fabry disease and its impact on women's lives.

## 5 Conclusions

There is a pressing need to provide tailored individual care for women living with Fabry disease and for more efforts to reduce societal prejudice. By fostering awareness, enhancing communication, and supporting healthcare professionals in their efforts to address both the medical and psychosocial aspects of the disease, we can improve the QoL for these patients. Future research should continue to explore the specific needs of women with Fabry disease and their caregivers, ensuring that their voices are heard and their experiences are understood. Such efforts will be instrumental in advancing patient care and reducing the burdens associated with this complex condition.

## CRediT authorship contribution statement

**Masahisa Kobayashi:** Writing – review & editing, Supervision, Methodology, Conceptualization. **Ikuko Kaku:** Writing – review & editing, Methodology, Investigation, Conceptualization. **Nanae Goto:** Writing – review & editing, Methodology, Investigation, Conceptualization. **Mio Tsuchiya:** Writing – review & editing, Methodology, Investigation, Conceptualization. **Norio Sakai:** Writing – review & editing, Supervision, Methodology.

## Funding

This study was funded by 10.13039/100015362Amicus Therapeutics, K.K., who provided financial support for the conduct of the research and preparation of the article, and supported with the study design and collection, analysis, and interpretation of data.

## Declaration of competing interest

The authors declare the following financial interests/personal relationships which may be considered as potential competing interests: Masahisa Kobayashi reports consulting fees from Amicus Therapeutics, K.K and payment or honoraria for lectures, presentations, speaker bureaus, manuscript writing or education events from Sanofi and Takeda Pharmaceuticals.

Ikuko Kaku has no conflicts of interest to report.

Nanae Goto reports employment with Amicus Therapeutics K.K.

Mio Tsuchiya reports previous employment with, and previous stockholder in, Amicus Therapeutics K.K.

Norio Sakai reports honoraria for lectures from Amicus Therapeutics K.K.

## Data Availability

Data will be made available upon request.

## Appendix A Supplementary data

Supplementary material Image 1
