# Supplementary material for: Exploring the burdens of women living with Fabry disease in Japan: A patient survey of 62 respondents
Source: Mol Genet Metab Rep. 2025 May 30;43:101231. doi: 10.1016/j.ymgmr.2025.101231 (PMC12163402; doi:10.1016/j.ymgmr.2025.101231)
Supplement: Supplementary file 1 — Supplementary material [file mmc1.docx]

# **Supplementary materials**

**Exploring the burdens of women living with Fabry disease in Japan: A patient survey of 62 respondents**

Masahisa Kobayashi, Ikuko Kaku, Nanae Goto, Mio Tsuchiya, Norio Sakai

**Fabry disease survey**

## Introduction (purpose of this study)

Symptoms of Fabry disease vary from person to person, but in women particularly, the manifestation and degree of symptoms vary greatly. Studies in other countries have shown that this makes early diagnosis and treatment more difficult for women than for men.

Overseas studies have also reported that quality of life for female patients is affected by life stages unique to women, such as pregnancy and childbirth, and, as with other genetic diseases, the possibility that the genetic changes that cause the disease may be transmitted to their children.

As described above, there are both medical and psychological issues that need to be examined in women living with Fabry disease, but no studies have been conducted in Japan that focus on the environment surrounding female patients.

For this reason, Amicus Therapeutics, Inc. in cooperation with Macromill Carenet, Inc. and the Japan Fabry Disease Patients and Family Association (JFA), has planned this survey to better understand the challenges of daily life for female patients with Fabry disease. The results obtained from this study will be widely shared with healthcare providers to help improve treatment for patients with Fabry disease.

## The subjects of this study

This study is open to participants who meet all of the following criteria:

1. Those who agree to participate in the research of their own free will.
2. Those who are at least 18 years of age at the time of obtaining consent.
3. Those who are able to respond to the web or paper survey in person.
4. People diagnosed with Fabry disease or caregivers/supporters of people diagnosed with Fabry disease.

## Questionnaire

**Q1** Have you received and understood the enclosed explanatory document?
[ ] Yes
[ ] No

**Q2:** Please tell us about your gender:
[ ] Male
[ ] Female

**Q3:** Please tell us about your age: ______ years old

**Q4:** Which of the following applies to your job (select one)?
[ ] Student
[ ] Company employee, public employee, or organization employee
[ ] Self-employed or freelance
[ ] Full-time homemaker
[ ] Part-time job
[ ] Unemployed
[ ] Other: ______________________________

**Q5:** Please select the response that best describes your relationship with Fabry disease (select one):
[ ] I am a patient (Proceed to Q7)
[ ] I am not only a patient but also a caregiver to other patient (Proceed to Q6)
[ ] I am a caregiver to other patient (Proceed to Q6)
[ ] Other: ______________________________ (Proceed to Q16)

**Q6:** You answered that you are a caregiver for someone living with Fabry disease in Q5. Who are you caring for? (Select all that apply)

[ ] Child

[ ] Spouse or partner

[ ] Parent

[ ] Other: ______________________________

*If you selected 1 or 2 in Q5, please answer the following questions. If you selected 3 or 4, please proceed to Q16.*

**Q7:** How old were you when you were you diagnosed with Fabry disease? ______ years old

**Q8:** Did you receive an explanation from your physician about Fabry disease and future prospects (select one)?
[ ] There was sufficient explanation
[ ] There was some explanation
[ ] There was not much explanation
[ ] There was no explanation at all
[ ] I don’t remember

**Q9:** Please tell us about your situation when you were diagnosed with Fabry disease (select one)
[ ] I was the first person among my family to be diagnosed
[ ] A family member was diagnosed first
[ ] Other: ______________________________

**Q10:** Do you currently have any symptoms related to Fabry disease?
[ ] Yes (Proceed to Q11)
[ ] No (Proceed to Q13)

**Q11:** Please tell us your current symptoms (select all that apply):
[ ] Pain in the hands or feet
[ ] No or reduced sweating
[ ] Hearing impairment
[ ] Gastrointestinal symptoms (stomach pain, diarrhea, etc)
[ ] Cardiac symptoms (enlarged heart, abnormal heart valves, arrhythmia, myocardial infarction, etc)
[ ] Renal symptoms (proteinuria, renal failure, dialysis, etc)
[ ] Cerebrovascular disorders (cerebral hemorrhage, subarachnoid hemorrhage)

**Q12:** Do you feel that you are able to share and communicate your symptoms sufficiently with your primary physician (select one)?

[ ] I think it is fully sufficient
[ ] I think it is okay
[ ] I can't say either way
[ ] I don’t think it is okay
[ ] I don’t think it is okay at all

**Q13:** Are you currently receiving specialized treatments for Fabry disease, such as enzyme replacement therapy (intravenous infusion) or chaperone therapy (oral medication)?
[ ] Yes, I am (Proceed to Q14)
[ ] I used to, but not anymore (Proceed to Q15)
[ ] No, I am not (Proceed to Q15)
[ ] I don’t know about treatment (Proceed to Q16)

**Q14:** Regarding your answer in Q13, which treatment are you receiving?
[ ] Enzyme replacement therapy (intravenous infusion)
[ ] Chaperone therapy (oral medication)

*If you answered Q14, please proceed to Q16.*

**Q15:** Regarding your answer in Q13, please tell us the reason (select all that apply)
[ ] I don’t have time because of work commitments
[ ] I don't have time because of family commitments
[ ] Financial burden (cost of medical care and transportation to the hospital)
[ ] I don't think it's a problem that needs treatment
[ ] I was told by my primary physician that there was no need for treatment
[ ] Communication with the primary physician is a psychological burden
[ ] Communication with the medical staff is a psychological burden
[ ] Other: ______________________________

**Q16:** Do you feel that the care that females receive for Fabry disease differs from that of males?

[ ] Yes
[ ] No

**Q17:** Please explain your answer to Q16:

____________________________________________________________________________________________________________________________________________

**Q18:** Which of the following are of most concern for women living with Fabry disease?* (Select all that apply)
*Women living with Fabry disease’ may include mothers, wives, daughters, or yourself as a patient or caregiver. Both men and women should answer the question about “women living with Fabry disease” from this point of view.

[ ] Fabry disease symptoms
[ ] Impact on quality of life
[ ] Path to diagnosis
[ ] Disease management
[ ] Treatment management
[ ] Inheritance of Fabry disease
[ ] Psychosocial wellbeing
[ ] The future of children with Fabry disease (education, employment, marriage, childbirth, etc)
[ ] Other (please specify): ______________________________

**Q19:** Please explain why you selected the biggest concern in the previous question?

____________________________________________________________________________________________________________________________________________

**Q20:** Which times in life is Fabry disease more of a concern for women living with Fabry disease? Please rank in order of priority:

| **Life stage** | **Rank** |
| --- | --- |
| Onset of puberty | ____ |
| Effect on the menstrual cycle | ____ |
| Planning a family, marriage, and childbirth | ____ |
| Perimenopause and menopause | ____ |
| Postmenopausal period | ____ |
| Formation of personal relationships (eg when entering school or finding a job) | ____ |
| Other: ______________________________ | ____ |

**Q21:** On a scale from 1 (strongly disagree) to 5 (strongly agree), please rank the following statements:

| **Statement** | **Strongly agree** | **Agree** | **Neither agree nor disagree** | **Disagree** | **Strongly disagree** |
| --- | --- | --- | --- | --- | --- |
| Primary physicians understand the unique challenges facing women living with Fabry disease | [ ] | [ ] | [ ] | [ ] | [ ] |
| Fabry specialists understand the unique challenges faced by women living with Fabry disease | [ ] | [ ] | [ ] | [ ] | [ ] |
| Women living with Fabry disease receive tailored individual care | [ ] | [ ] | [ ] | [ ] | [ ] |
| My family or the colleagues I work with understand the unique challenges faced by women living with Fabry disease | [ ] | [ ] | [ ] | [ ] | [ ] |
| Women living with Fabry disease are recognized by the patient organizations I belong to | [ ] | [ ] | [ ] | [ ] | [ ] |
| Fabry disease affects the mental health of women living with Fabry disease | [ ] | [ ] | [ ] | [ ] | [ ] |

**Q22:** In your opinion, what is the most significant unmet need for women living with Fabry disease?

______________________________________________________________________
